# The Great Mimicker Gets Caught: A Rare Case of Syphilis in the Gastrointestinal Tract

**DOI:** 10.7759/cureus.59222

**Published:** 2024-04-28

**Authors:** Carlos Cantu Lopez, Sarahi Herrera-Gonzalez, Dema Shamoon, Theodore Jr Dacosta, Yatinder Bains

**Affiliations:** 1 Internal Medicine, Saint Michael's Medical Center, Newark, USA; 2 Gastroenterology and Hepatology, Saint Michael's Medical Center, Newark, USA

**Keywords:** sexually transmitted diseases, syphilis, rectal mass, elevated liver function test (lfts), infectious hepatitis, syphilitic hepatitis, syphilitic proctitis

## Abstract

Syphilis is a sexually transmitted disease that impacts multiple organ systems and can mimic various diseases and is an extremely rare cause of proctitis in men who have sex with men and transgender females. We present a case of a 49-year-old transgender female with a medical history significant for diabetes mellitus and hyperlipidemia who presented to the emergency department with dull abdominal pain in the left upper and lower quadrants for two days. She had non-bloody, nonbilious emesis, 10-pound weight loss over 1 month, and constipation for 2 weeks. Laboratory results showed a cholestatic pattern. Computed tomography of the abdomen showed rectal wall thickening, multiple enlarged perirectal adenopathy, and mild inflammatory infiltration around the rectum suggesting superimposed proctitis. On colonoscopy, a possible rectal mass or severe proctitis with near complete obstruction was seen with initial pathology concerning for lymphoma or a rare type of colitis. The patient was empirically started on ceftriaxone and doxycycline leading to improvement in inflammation. Special stains requested were positive for Treponema pallidum confirming the diagnosis of syphilitic proctitis and highly suggestive syphilitic hepatitis. Few cases of syphilitic proctitis imitating rectal malignancy and syphilitic hepatitis have been reported. Syphilis requires exclusion as well as confirmation of spirochetes for high-risk populations with special staining. It is important to diagnose syphilis in special populations that are at high risk of contraction.

## Introduction

Syphilis is a sexually transmitted disease (STD) that presents in a vast number of organ systems. It has been called the “great mimicker,” as it may resemble many other pathologies, therefore, it is important to have a high degree of suspicion in patients with high-risk sexual practices.

Syphilitic proctitis represents only 1% of sexually transmitted proctitis in men who have sex with men (MSM) and transgender females [1] while syphilitic hepatitis has a described incidence of 0.25% to 38% [2]. This is a rare case of syphilis presenting in the gastrointestinal tract (GIT) with simultaneous hepatitis and biopsy-proven proctitis, representing the first case reported in the literature since 2011 [3].

This article was previously presented as a meeting abstract at the 2022 American College of Gastroenterology Annual Scientific Meeting and Postgraduate Course on October 23, 2022.

## Case presentation

A 49-year-old transgender female, with a medical history significant for diabetes mellitus and hyperlipidemia, presented to the emergency department, complaining of dull abdominal pain located in the left upper and lower quadrants for two days and associated with multiple episodes of non-bloody, non-bilious emesis. On review of systems, she had unintentional 10-pound weight loss in the last months and a two-week history of constipation with mucous and small-volume bowel movements. She denied fever, dysuria, and rectal bleeding. Laboratory results were significant for a sodium level of 129 (136-145 mmol/L), aspartate aminotransferase (AST) of 397 (10-36 U/L), alanine aminotransferase (ALT) of 224 (6-46 U/L), alkaline phosphatase (ALP) of 889 (33-130 U/L), and total bilirubin of 7.3 (0.2-1.2 mg/dL). Cell counts were all unremarkable. Subsequent testing showed carbohydrate antigen 19-9 of 125.2 (0.0-34 U/mL) and a carcinoembryonic antigen 3.0 (0.0-3.0 ng/mL (Table 1).

**Table 1:** Laboratory investigations on presentation

Blood Chemistry	Value	Reference Range
Sodium	129	136 - 145 mmol/L
Chloride	94	90 - 110 mmol/L
Aspartate Transaminase (AST)	397	10 - 36 U/L
Alanine Transaminase (ALT)	224	6 - 46 U/L
Alkaline Phosphatase (ALP)	889	33 - 130 U/L
Total Bilirubin	5.7	0.2 - 1.2 mg/dL
Albumin	3.1	3.6 - 5.1 g/dL
Gamma GT (GGT)	1170	3 - 85 U/L
White blood cell (WBC)	6.60	4.40 - 11.0 10*3/uL
Hemoglobin	15.1	13.5 - 17.5 g/dL
Hematocrit	44.7	38.8 - 50.0%
Platelets	350	150 - 450 10*3/uL
Actin smooth muscle antibody (ASMA)	15	0 - 19 units
Anti-mitochondrial antibody (AMA)	23.7	Negative: 0.0 - 20 units; Equivocal: 20.1 - 24.9 units; Positive: > 24.9 units
Anti-nuclear antibody (ANA)	Negative	Negative
Immunoglobulin G (IgG)	1220	767 - 1590 mg/dL
Perinuclear (p-ANCA)	<1:20	Negative: <1:20 titer
Carbohydrate antigen 19-9 (CA19-9)	125.2	0.0 - 34.0 U/mL
Carcinoembryonic antigen (CEA)	3.0	0.0 – 3.0 ng/mL
Rapid plasma reagin (RPR)	Reactive	Non-reactive
RPR titer	1:4	None
Hepatitis A total antibody	Reactive	Non-reactive
Hepatitis A IgM antibody	Non-reactive	Non-reactive
Hepatitis B surface antigen	Non-reactive	Non-reactive, equivocal
Hepatitis B core IgM	Non-reactive	Non-reactive
Hepatitis B surface antibody	Reactive	Non-reactive
Hepatitis C antibody	Non-reactive	Non-reactive
HIV 1&2 antibody	Non-reactive	Non-reactive

**Figure 1:**
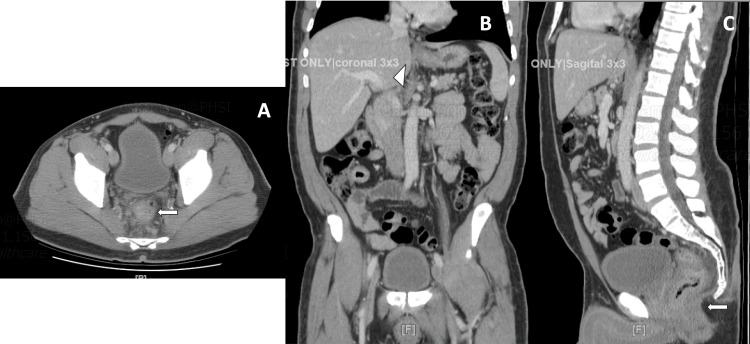
CT of abdomen and pelvis Images showing hepatic steatosis with a normal caliber of the biliary system (arrowhead), focal moderate wall thickening of the rectum (arrows), and multiple enlarged perirectal adenopathy (A. Axial; B. Coronal; C. Sagittal)

Computed tomography (CT) of the abdomen and pelvis with IV contrast showed hepatic steatosis with the normal caliber of the biliary system, focal moderate wall thickening of the rectum with multiple enlarged perirectal adenopathy concerning for a rectal mass, as well as mild inflammatory infiltration around the rectum suggesting superimposed proctitis (Figure 1).

Our differential diagnoses consisted of infectious proctitis, viral hepatitis, primary biliary cholangitis (PBC), ulcerative colitis with primary sclerosing cholangitis, and, of course, rectal malignancy with metastatic liver disease.

**Figure 2:**
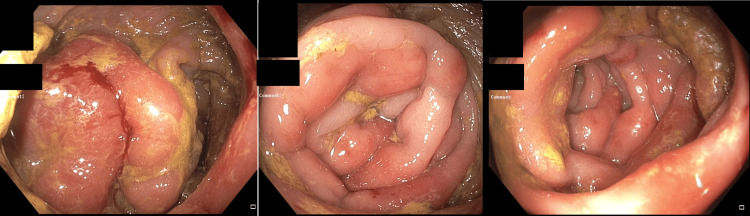
Colonoscopy images Images of initial colonoscopy showing severe inflammation, erythema, and thickened rectal folds with near luminal obstruction prior to the initiation of syphilis treatment (blacked out identifying patient information in the left upper corner of images).

A colonoscopy was performed the following day, which revealed a possible rectal mass versus severe proctitis with erythema and inflammation with near complete obstruction which was unable to be traversed (Figure 2).

**Figure 3:**
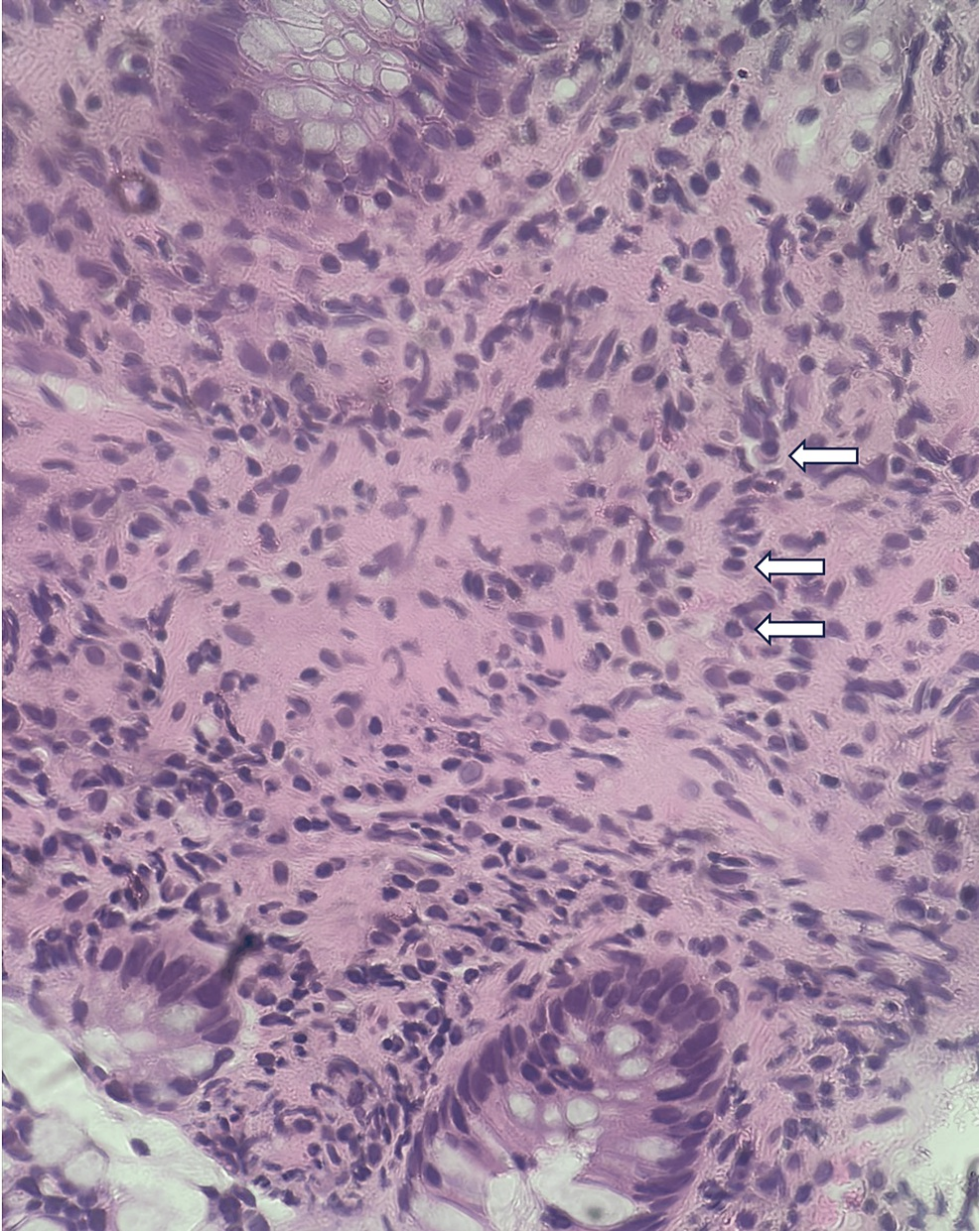
Hematoxylin and eosin stain of colon biopsies Pathology image of colonic mucosa with marked expansion of the lamina propria by mixed neutrophilic and lymphoplasmacytic infiltrate (arrows), suggestive of lymphoma or a very rare form of colitis

Initial pathology demonstrated a possible lymphoma versus a very rare type of colitis (Figure 3).

Immunohistochemistry would later rule out lymphoma. Magnetic resonance cholangiopancreatography (MRCP) was unremarkable. Anti-smooth muscle antibody (ASMA), anti-nuclear antibody (ANA), immunoglobulin G (IgG), and perinuclear anti-neutrophil cytoplasmic antibodies (p-ANCA) were negative while anti-mitochondrial antibody (AMA) was equivocal at 23.7 (negative <20 Units, equivocal 20.1-24.9 and positive > 24.9) (Table 1). The viral hepatitis panel showed immunity to Hepatitis A and B, and negative Hepatitis C. Human immunodeficiency virus (HIV) was also negative. The patient was empirically started on ceftriaxone and doxycycline while waiting for an infectious proctitis workup for syphilis, chlamydia, and gonorrhea.

**Figure 4:**
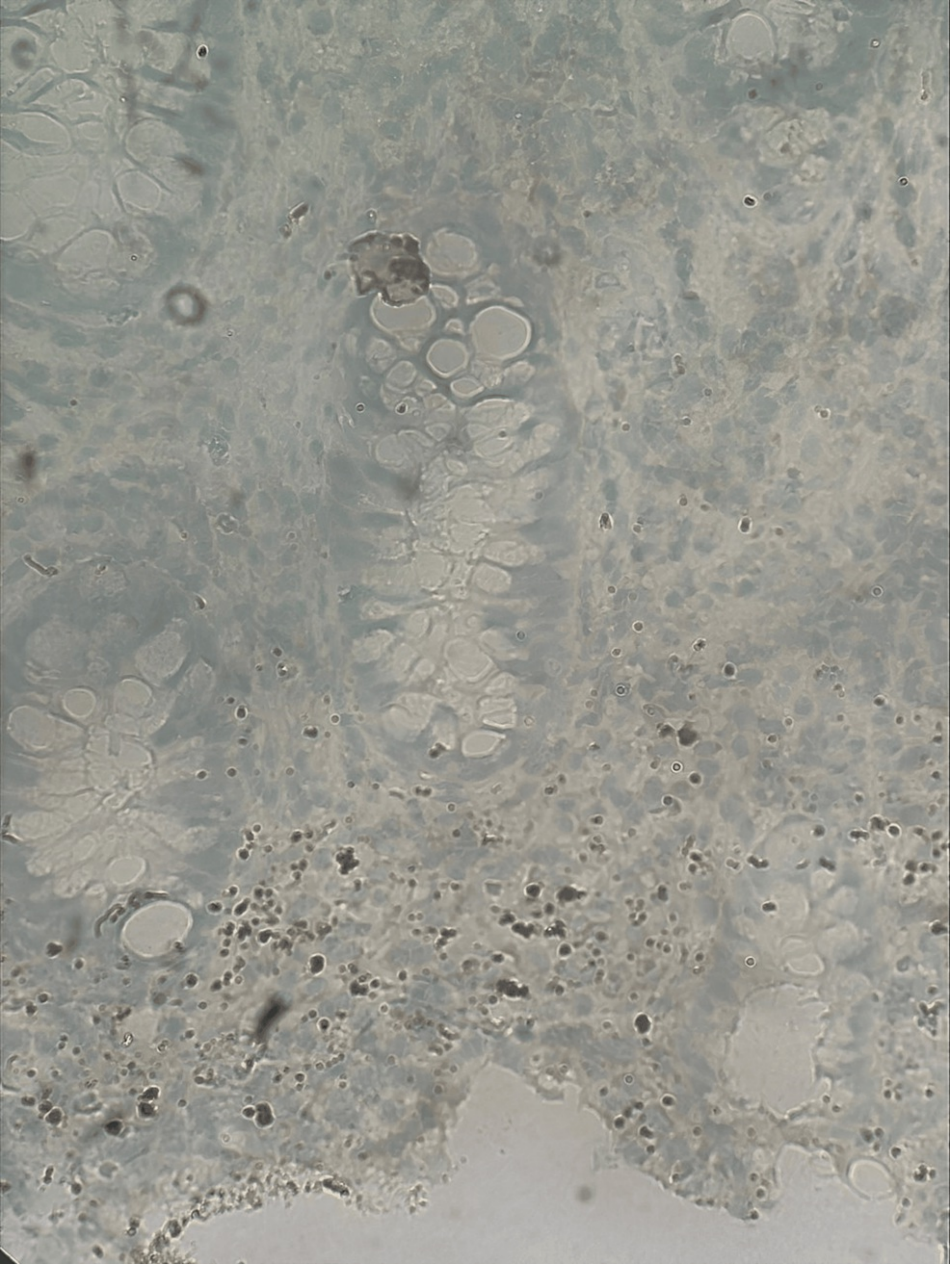
Rectal biopsy with a positive Treponema pallidum stain by immunohistochemistry

A second colonoscopy was performed four days later, and although still present, a significant decrease in overall inflammation was noted. Colonoscopy was otherwise grossly unremarkable proximal to the rectum. Chlamydia and gonorrhea tests were both negative, while rapid plasma reagin (RPR) was weakly positive at 1:4 titer. A fluorescent treponemal antibody-absorption test was sent for confirmation and was positive. One month prior to the presentation, all labs including RPR were negative. Special stains were requested and resulted in positive for Treponema pallidum (Figure 4) and negative for human Herpesvirus-8 (HH8), confirming the diagnosis of syphilitic proctitis and highly suggestive syphilitic hepatitis. Prior to discharge, transaminases and bilirubin were significantly decreased. She received a one-month course of doxycycline with a resolution of symptoms.

## Discussion

Syphilis is the cause of sexually transmitted proctitis in only 1% of MSM and transgender females [1]. Diagnosis requires a high index of suspicion in populations who engage in high-risk sexual behaviors. Given that spirochetes are not seen on pathology specimens with routine hematoxylin and eosin stains, special staining for spirochetes must be explicitly requested of the pathologist [4].

Syphilitic proctitis may mimic rectal tumors on imaging and endoscopy, as well as clinical presentation such as in our patient. Bronson et al. (2021) described a case of rectal syphilis presenting with a palpable mass and imaging studies showing rectal wall thickening and enlarged pelvic lymph nodes [5]. Initial pathology was suggestive of lymphoma but was subsequently ruled out by immunohistochemistry. Finally, the diagnosis of syphilitic proctitis was made by positive spirochetes staining [5]. A few other cases of syphilitic proctitis imitating rectal malignancy have been reported [6,7]. Treatment is with benzathine penicillin G, and doxycycline or tetracycline for patients with penicillin allergy [8].

Syphilitic hepatitis is also very rare, with priorly reported incidence ranging between 0.25% and 38% [2]. It presents mostly with elevation of liver function tests (LFTs) in a cholestatic pattern. Presents by causing intrahepatic cholestasis with normal biliary tract anatomy on imaging. Tissue diagnosis is not generally required and may be done by a combination of abnormal LFTs, serological evidence of syphilis, exclusion of other causes of liver disease, and normalization of LFTs after treatment (all four criteria required) [9]. When histopathologic samples are obtained, commonly seen findings include hepatic granulomas and biliary ductal inflammatory infiltration [10].

A case of syphilis hepatitis mimicking PBC was described by Kern et al. In their case, a positive AMA was seen, which also turned negative after treatment for syphilis [11]. While our patient had an equivocal result, this was likely elevated due to syphilis involvement of the liver.

## Conclusions

Syphilis is an uncommon yet potentially severe cause of pathology in the GIT. It most frequently presents impersonating serious causes of pathology. It is imperative to perform careful evaluation and proper history-taking in high-risk populations, with particular attention to sexual history. Like in our patient’s case, recognizing that multiple organ systems may be simultaneously involved may facilitate the prompt diagnosis and management of this great mimicker.
